# Analysis of the association between dietary sodium intake and cognitive function: a NHANES-based machine learning study and animal experimental validation

**DOI:** 10.3389/fnut.2025.1626651

**Published:** 2025-09-08

**Authors:** Shuyu Wang, Kejie Deng, Hongyi Zhao, Yi Wang, Pei Guo, Hongjiao Jin, Luming Qi

**Affiliations:** ^1^School of Health Preservation and Rehabilitation, Chengdu University of Traditional Chinese Medicine, Chengdu, China; ^2^Acupuncture Rehabilitation Center, Hospital of Chengdu University of Traditional Chinese Medicine, Chengdu, China; ^3^School of Basic Medical Sciences, Chengdu University of Traditional Chinese Medicine, Chengdu, China; ^4^Department of Pediatrics, The Third Affiliated Hospital of Zunyi Medical University, The First People's Hospital of Zunyi, Zunyi, China

**Keywords:** sodium intake, cognitive function, NHANES, machine learning, animal behavior

## Abstract

**Background:**

Sodium intake is undoubtedly essential for normal bodily function, but it is an important public health concern when intake exceeds dietary requirements. With salt intake exceeding recommended levels in almost all countries, high salt intake has become an important global health topic. This study aimed to be the first to combine epidemiological big data with animal behavioral experiments to systematically investigate the association between dietary sodium intake and cognitive function.

**Methods:**

Based on NHANES data from 2011 to 2014, the association between dietary sodium intake and cognitive impairment was assessed using multivariate linear regression and five types of machine learning, including random forest and XGBoost, in 2367 participants aged 60–80 years. Cognitive function was further assessed by a 6-month high-salt dietary intervention in a C57BL/6 mouse model combined with behavioral tests such as the Morris water maze and Open Field Test (OFT).

**Results:**

The results of the study revealed that the average daily sodium intake of the 2,367 middle-aged and older adults included reached 4,502 mg, with 91% exceeding the recommended standard. Dietary sodium intake was positively correlated with cognitive function scores (*p* < 0.001). The random forest model had the best predictive efficacy (AUC = 0.918), which was significantly better than that of the SVM and neural networks. Animal behavioral experiments revealed that high**-**salt dietary exposure significantly impaired neurobehavioral function in a dose-dependent manner, leading to spatial memory deficits and anxious behavior in mice.

**Conclusion:**

There was a threshold-dependent association between dietary sodium intake and cognitive function, and the dose–response trend was consistent between human observational studies and animal studies. This study provides translational medical evidence for the public health strategy of “salt reduction for dementia prevention.

## 1 Introduction

Cognitive dysfunction, a clinical syndrome characterized by the progressive impairment of core cognitive domains such as memory, attention, and executive function, has become an important public health challenge in aging societies worldwide as a precursor stage of dementia (1, 2). Patients with cognitive impairment experience difficulties in basic tasks of daily living, leading to reduced quality of life and increased mortality, with a substantial economic and social burden (3). According to the latest data released by the WHO, the number of people with dementia is expected to exceed 150 million worldwide by 2050, and 30% of cases could be prevented through intervenable risk factors (4). In this context, the critical role of modifiable environmental factors, particularly dietary patterns, in modulating cognitive function should be explored (5).

Sodium is a nutrient that is essential for the maintenance of human health and physiology, and sodium ions are involved in central nervous system regulation through physiological processes such as the maintenance of cellular osmotic pressure and nerve conduction (6). However, excessive sodium intake, which is prevalent in modern diets, may produce neurotoxic effects (7). Animal experiments have confirmed that high-sodium diets may impair cognitive functions such as spatial memory by inducing cerebrovascular endothelial dysfunction, blood–brain barrier disruption, and neuroinflammatory responses, accelerating Aβ protein deposition and tau protein hyperphosphorylation (8–10). However, population studies present conflicting findings. In an analysis limited to high-quality studies, three-quarters reported that higher sodium intake was associated with impaired cognitive function (11–14). These inconsistent findings may be due to variations in study populations, small sample sizes, or other confounding factors. Because of these confounding factors, it is difficult for observational studies to determine the independent effect of salt intake on dementia risk.

The National Health and Nutrition Examination Survey (NHANES) is a research program designed to assess the health and nutritional status of adults and children in the United States. The survey combines interviews and physical examinations, focuses on a wide range of health and nutrition indicators, and provides data on a broad range of topics, including socioeconomics, diet, health status, demographics, and laboratory testing. This study investigated the relationship between dietary sodium intake and cognitive dysfunction using data from the NHANES from 2011 to 2014. The study assessed dietary sodium intake via a standardized 24-h dietary recall method combined with validated cognitive assessment tools such as the Digit Symbol Substitution Test (DSST) and the Coalition of Alzheimer’s Disease Registries (CERAD WL). It also included data on multidimensional covariates, such as demographic characteristics, and multivariate logistic regression analyses were performed to explore these associations, with adjustments for key covariates. Subgroup analyses were conducted to ensure robustness across populations. Previous NHANES-based studies have used linear regression analyses, which may overlook the complex nonlinear relationships between nutrient–health outcomes and interactions between variables. In addition, this study innovatively integrated machine learning models to explore more complex patterns and quantify the relative contribution of sodium intake in predictive models of cognitive disorders, thereby further understanding the potential metabolic contribution of these diseases. The results of this study will further shed light on the scientific implications of high-salt diet-induced cognitive impairment, rationalize salt intake in terms of its importance, and have important implications for the development of strategies to support cognitive health and prevent cognitive decline in older adults.

## 2 Materials and methods

### 2.1 Sample populations in the NHANES

**Figure 1:**
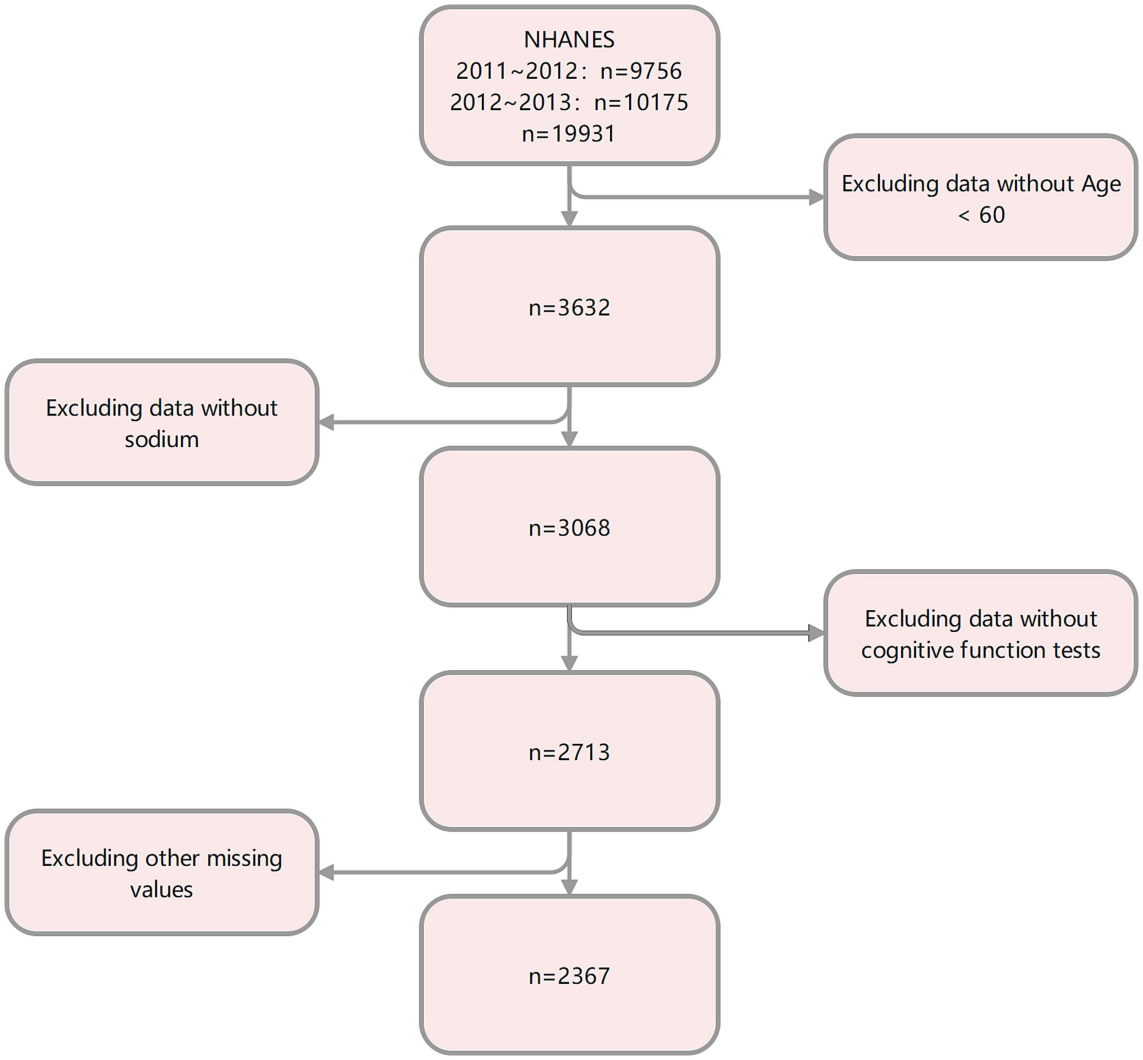
Selection of the study population.

The data used in this study were obtained from the 2011–2014 National Health and Nutrition Examination Survey (NHANES) dataset, available at https://www.cdc.gov/nchs/nhanes/. The National Health Survey (NHANES) is conducted by the Centers for Disease Control and Prevention (CDC) and the National Center for Health Statistics (NCHS). It was designed to analyze the health and nutritional status of a nationally representative, noninstitutionalized sample of U.S. citizens. Participants aged 60–80 years with available information on dietary sodium intake and who had undergone a complete cognitive function test were recruited for this study. After excluding subjects with missing information on covariates, a final cohort of 2,367 individuals was included in this study. The statistical methods used in this study were cross-sectional and followed the STROBE guidelines. Table 1 summarizes the basic characteristics of the participants in the dietary sodium intake quartiles. A specific flowchart is shown in Figure 1. The NCHS Research Ethics Review Board approved the investigation protocol, and all participants provided written informed consent.

**Table 1:** Basic characteristics of participants in the `DSST_Quartile’.

Variable	D1	D2	D3	D4	*p*-overall
	*N = 553*	*N = 574*	*N = 620*	*N = 620*	
Age					<0.001
60 ~ 80	553		435	335	
Gender					<0.001
Male	322 (56.0%)	327 (55.6%)	304 (48.4%)	229 (36.1%)	
Female	253 (44.0%)	261 (44.4%)	324 (51.6%)	405 (63.9%)	
Race					<0.001
Mexican American	72 (12.5%)	62 (10.5%)	39 (6.21%)	36 (5.68%)	
Other Hispanics	97 (16.9%)	56 (9.52%)	45 (7.17%)	33 (5.21%)	
Non-Hispanic whites	176 (30.6%)	288 (49.0%)	363 (57.8%)	413 (65.1%)	
Non-Hispanic blacks	199 (34.6%)	135 (23.0%)	118 (18.8%)	73 (11.5%)	
Other races	31 (5.39%)	47 (7.99%)	63 (10.0%)	79 (12.5%)	
Marital					<0.001
Unmarried	83 (14.4%)	91 (15.5%)	105 (16.7%)	118 (18.6%)	
Married	314 (54.6%)	368 (62.6%)	404 (64.3%)	427 (67.4%)	
Widowed/divorced	178 (31.0%)	129 (21.9%)	119 (18.9%)	89 (14.0%)	
EDU					<0.001
Lower than high school	186 (32.3%)	49 (8.33%)	14 (2.23%)	4 (0.63%)	
Congrats! (on passing an exam)	137 (23.8%)	114 (19.4%)	65 (10.4%)	19 (3.00%)	
High school and above	252 (43.8%)	425 (72.3%)	549 (87.4%)	611 (96.4%)	
BMI					0.564
Normal weight	156 (27.1%)	148 (25.2%)	166 (26.4%)	166 (26.2%)	
Overweight (baggage, freight)	208 (36.2%)	218 (37.1%)	205 (32.6%)	236 (37.2%)	
Obese	211 (36.7%)	222 (37.8%)	257 (40.9%)	232 (36.6%)	
Smoke					<0.001
Cigarette smoking	86 (15.0%)	73 (12.4%)	75 (11.9%)	42 (6.62%)	
Used to smoke	213 (37.0%)	253 (43.0%)	246 (39.2%)	239 (37.7%)	
Nonsmoking	276 (48.0%)	262 (44.6%)	307 (48.9%)	353 (55.7%)	
Hypertension					<0.001
Clogged	165 (28.7%)	218 (37.1%)	230 (36.6%)	290 (45.7%)	
Be	410 (71.3%)	370 (62.9%)	398 (63.4%)	344 (54.3%)	
Sodium_Quartile					<0.001
S1	238 (41.4%)	161 (27.4%)	167 (26.6%)	146 (23.0%)	
S2	163 (28.3%)	174 (29.6%)	190 (30.3%)	175 (27.6%)	
S3	109 (19.0%)	142 (24.1%)	177 (28.2%)	191 (30.1%)	
S4	65 (11.3%)	111 (18.9%)	94 (15.0%)	122 (19.2%)	

### 2.2 Variables

#### 2.2.1 Dietary sodium intake

Dietary sodium was assessed by conducting either a 24-h dietary recall interview at a mobile examination center or by telephone. For the 2011–2014 surveys, two 24-h dietary recall interviews were conducted: the first in person at a mobile exam center and the second by telephone 3–10 days later. For both cycles, dietary sodium averages calculated from the two interviews were used in the analyses. The 24-h recall data were collected via the U.S. Department of Agriculture’s Dietary Data Collection Instrument, which uses the Dietary Research Food and Nutritional Database to convert a typical portion of food into grams of total nutrient intake. The dietary sodium data from the seven NHANES were post-adjusted for the amount of salt used when preparing food at home based on answers to the following question: ‘How often is regular or seasoned salt added when cooking or preparing food at home?’ Is it never, rarely, occasionally, or very often?” If “rarely” or “never,” we removed all optional salt; if “occasionally,” we removed half of the optional salt (15). The dietary sodium density was calculated as total sodium (mg) divided by total dietary energy (kcal).

To address recall bias and reporting errors in NHANES 24-h dietary recalls, the survey implemented the validated Automated Multiple-Pass Method (AMPM) for both in-person and telephone interviews. This protocol ensured proportional coverage across all days of the week and seasons to minimize random variation. Biomarker verification, including doubly labeled water measurements and 24-h urinary excretion analyses, was employed to validate intake data. Statistical models incorporating external datasets corrected systematic underestimation. Additionally, rigorous interviewer training, computerized data collection tools, and multiple non-consecutive recall days with measurement error adjustment substantially enhanced the accuracy of population-level dietary estimates (16).

#### 2.2.2 Cognitive dysfunction

The cognitive function assessment consists of three different tests. First, the digit symbol substitution test (DSST) assesses sustained attention, psychomotor speed, and working memory. The participants were required to correctly match a series of symbols with the corresponding numbers within 2 min. According to the DSST scale, participants receive a score based on the number of symbols correctly matched, with a maximum score of 133, with higher scores indicating better cognitive functioning (17). Second, the Alzheimer’s Disease Registry Consortium (CERAD WL) assesses an individual’s ability to learn new language material. The participants were required to recall a set of 10 unrelated words over three learning attempts, and a delayed recall test was administered approximately 10 min later. A total score of 40 reflects learning and memory skills (18). Finally, the animal fluency test assesses language category fluency and executive function. The participants were asked to list the names of as many animals as possible in 1 min to assess semantic memory and processing speed (19).

#### 2.2.3 Covariate information

All participants provided information via questionnaires about age, sex, race (categorized as Mexican American, other Hispanic, non-Hispanic white, non-Hispanic black, or other races), marital status (categorized as unmarried, married, or celibate), education (categorized as less than high school, high school, or above), smoking status (categorized as a smoker, ex-smoker, or never-smoker), hypertension, and body mass index (BMI), which were calculated as weight (kg)/height (m^2^).

### 2.3 Machine learning

To further elucidate the relationship between dietary sodium intake and cognitive impairment, we introduced machine learning to verify this association. The primary process was carried out using the sklearn, PyTorch, and NumPy libraries in Python. Four different machine learning models were used in this study: XGBoost (extreme gradient boosting), random forest, LightGBM (light gradient boosting), SVM (support vector machine), and a fully connected deep neural network. To evaluate the results of these three classification models, we compare the area under the curve (AUC), accuracy, and F1 score. By default, the best model is selected based on the highest AUC.

### 2.4 Animal experiments

#### 2.4.1 Laboratory animals

Eighteen healthy C57BL/6 male mice, aged 3 months and weighing 22 ± 5 g, were born at the same time and bred in the same batch under the same conditions. This project was conducted at the Animal Experiment Center of Chengdu University of Traditional Chinese Medicine. The room temperature was controlled at 18–22 °C, the relative humidity was maintained at 60–70%, and the light exposure period was 12 h:12 h (light:dark).

#### 2.4.2 Animal model establishment and grouping

After 1 week of adaptive feeding, 18 healthy C57BL/6 rats (males, 6–8 weeks old) were weighed, numbered, and grouped. Six were selected as a normal control group (ND) using the random number method. They were given a normal diet and free drinking water for 6 months. Six were in the high-salt diet group 1 (HSD1), who were given a normal diet for 3 months, followed by a high-salt diet (8% NaCl) for 3 months, and Ad libitum drinking water. Six were in a high-salt diet group 2 (HSD2); they were given a high-salt diet (8% NaCl) for 6 months and drank free water.

The high-salt formulation contained (w/w): 18% moisture, 50% crude protein, 9% crude fat, 4% crude fiber, 6% ash, 3% calcium, 2% total phosphorus, and 8% NaCl.

#### 2.4.3 Behavioral experiments

##### 2.4.3.1 Morris water maze experiment

The Morris water maze test was used to test the learning and memory ability of the mice after 6 months of a high-salt diet.

A circular pool with a diameter of 1.2 m and a depth of 0.4 m was fabricated in the water maze apparatus. The temperature inside the pool was maintained at 25 ± 1 °C throughout the study period. The pool was divided into four equal quadrants (I, II, III, and IV), and in quadrant I, a hidden circular platform with a diameter of 8 cm was located 1 cm below the surface of the water. A video camera was mounted above the center of the labyrinth to record the movements of the mice (*n* = 6/group). These mice were trained for 1 min three times per day for four consecutive days. During each session, the mice were placed in water and allowed to swim freely for 60 s. If a mouse succeeded in finding and climbing up to the hidden platform, it was given a 30-s rest period and then removed from the pool. If a mouse did not find the platform within the designated time, it was gently guided to it and remained there for 30 s. On day 5, a spatial memory detection experiment was conducted. In this experiment, the platform was removed, and the mouse was placed in the quadrant opposite quadrant I. The mouse was given 60 s to swim. The mice were given 60 s to swim, and the number of times they crossed the platform within 60 s, the duration and distance of the swimming in the quadrant where the platform was located, and the average swimming speed were analyzed to assess their spatial memory ability. All the data were recorded via a MORRIS water maze video tracking system (Taimeng, Chengdu, Model: WMT-100S).

##### 2.4.3.2 Absent field experiment

The open field test (OFT) is a classical behavioral test that assesses the anxiety level of experimental animals. The test measures the ability of a mouse to move independently in an open environment and the time spent in the center of the open field. The mouse OFT device is 30 cm high, 50 cm long, and broad at the bottom, with a white inner wall. It is artificially divided into 16 compartments, with four compartments in the inner area and 12 compartments in the outer. The experimental site was quiet to avoid sound stimulation of the mice, which affected the accuracy of the experimental results. The mice were placed at the center of the bottom of the box while the camera and timing were performed. The camera’s field of view covered the entire open field area and recorded the mice’s voluntary movement and the number of times they traveled between the squares. The camera was stopped after a specific period of observation, which lasted 5 min, and the inside and bottom of the box were wiped with 75% alcohol to prevent the residual feces and odor of the animal from affecting the results of the next test animal. The procedure was repeated after the mice were replaced until all the mice had completed the test.

### 2.5 Statistical analysis

We used Python 3.10 as the data analysis tool, adhering to the NHANES analysis guidelines. Parameters such as sample weights, stratification, and primary sampling units were applied to the complex survey design to ensure that the data accurately reflected the U.S. noninstitutionalized population. For continuous variables, the overall characteristics of the studies are expressed as the means (SE) or percentages. To detect potential associations between dietary sodium intake and cognitive dysfunction, we used weighted linear regression analyses with calculated *β* values and 95% confidence intervals (CIs). Next, we sequentially modeled the three included covariates with additional adjustments. Multiple linear regression was used to analyze the cognitive function assessment scores. Three different models were fitted: Model 1, without covariates, was adjusted; Model 2 included sex, age, race, and marital status. Model 3 was adjusted for education, BMI, hypertension, and variables from Model 2.

The mice were randomized via the RANDBETWEEN function in Microsoft Excel to generate random numbers for the allocation of the mice. All experimental data in this study are expressed as the mean ± standard deviation (SD). Data analysis was performed via SPSS 25.0 and GraphPad Prism 9.5 statistical software. Before statistical analysis, the data in each group were first tested for normality (Shapiro–Wilk test) and chi-square test (Levene test). One-way ANOVA was used for data that were normally distributed and passed the test for homogeneity of variance (e.g., Levene’s test). If the ANOVA results were statistically significant, multiple comparisons were further performed using the Least Significant Difference (LSD) method. Statistical significance was set at *p < 0.05, **p < 0.01, and ***p < 0.001.

## 3 Results

### 3.1 General characteristics of NHANES

The age range of the participants in the study was 60–80 years, with a total of 2,367, of which 1,182 were males and 1,243 were females. Table 1 summarizes the basic characteristics of the study population. The mean daily dietary sodium intake of all participants was 4,502 mg, which was higher in 91% of the participants (94% of females and 88% of males) than the general recommendation of 2,300 mg/day. The dietary sodium intake quartiles were significantly different (*p* < 0.05) for the variables of age, sex, race, marital status, education, smoking, hypertension, and cognitive function test scores. However, no statistically significant differences were observed for BMI (*p* > 0.05).

### 3.2 Relationship between dietary sodium intake and cognitive dysfunction

Weighted linear regression analysis was used to explore the association between dietary sodium intake and cognitive dysfunction. Table 2 shows the significance (*p*-value) of the associations of different cognitive function tests with dietary sodium intake under different statistical models. The DSST showed a highly significant association in all the models (*p* < 0.001), suggesting that the potential effect of sodium intake on processing speed is stable and may be free from confounding factors. The significance of the animal fluency test remained. However, it decreased slightly with model adjustment (p rose from <0.001 to 0.005), suggesting that the association between sodium intake and verbal fluency may exist independently.

**Table 2:** Association between dietary sodium and cognitive dysfunction among adults.

*p* value			
Depression and cognitive dysfunction scores	Crude model (Model 1)	Minimally adjusted model (Model 2)	Fully adjusted model (Model 3)
DSS	<0.001	<0.001	<0.001
CREAD total word recall	0.023	0.012	0.233
CREAD delayed recall	0.057	0.041	0.078
Animal fluency test	<0.001	0.001	0.0

However, the results of CERAD WL indicated that immediate recall (total word recall) was significant in the unadjusted and partially adjusted models but lost significance after full adjustment (*p* = 0.233); delayed recall was only transiently significant in the partially adjusted model (*p* = 0.041) and was not significant in the other models. These findings suggest that the association between sodium intake and memory function may be partially mediated by confounding variables such as education, BMI, and chronic disease rather than a direct effect. Taken together, the effects of dietary sodium intake on information processing speed are strongly robust, whereas the effects on memory function can be mediated by factors such as education level and chronic disease, and the reason for this heterogeneity suggests that sodium ions may act on specific cognitive domains through different physiological pathways.

In this study, we excluded samples outside the most extreme 2.5% ~ 97.5% quantile, performed a sensitivity analysis (20), and found that the direction and significance of the main covariates were highly consistent with those of the main model, indicating that the results were not sensitive to extreme sodium intake values and that the conclusions were robust. The results are presented in Supplementary Table 1.

### 3.3 Machine learning results

We deployed various machine learning models, including XGBoost (Extreme Gradient Boosting), Random Forest, LightGBM (Light Gradient Boosting Machine), SVM (Support Vector Machine), and Fully Connected Deep Neural Network. The patient’s degree of cognitive dysfunction was dichotomized and predicted based on the patient’s DSST score. Based on the performance comparison of the machine learning models in Table 3, the random forest has an AUC of 0.92, which is the best among the five machine learning algorithms, followed by XGBoost and LightGBM, with AUCs of 0.89 and 0.85, respectively; these scores are better than those of the SVM and the neural network. The higher AUC values indicate that random forest has a stronger integrative ability in distinguishing between positive and negative class samples, possibly because of its robustness to high-dimensional data and its ability to capture feature interactions automatically. In contrast, the SVM has the weakest performance in terms of both metrics, which may be related to its lack of adaptability to high-dimensional data or its high tuning complexity. Neural networks perform moderately well, implying that, although they have some potential in the current task, they may be limited by data size or structural characteristics. The ROC curves in Figure 1 show the same results.

**Table 3:** Results of the machine learning model: Acc and AUC.

Model	*Acc*	AUC	F1-score	95%CI
Xgboost	0.82	0.89	0.83	(0.86, 0.91)
Random Forest	0.83	0.92	0.83	(0.90, 0.94)
LightGBM	0.81	0.88	0.81	(0.86, 0.90)
SVM	0.73	0.80	0.73	(0.77, 0.83)
Neural Network	0.78	0.85	0.78	(0.82, 0.87)

**Figure 2:**
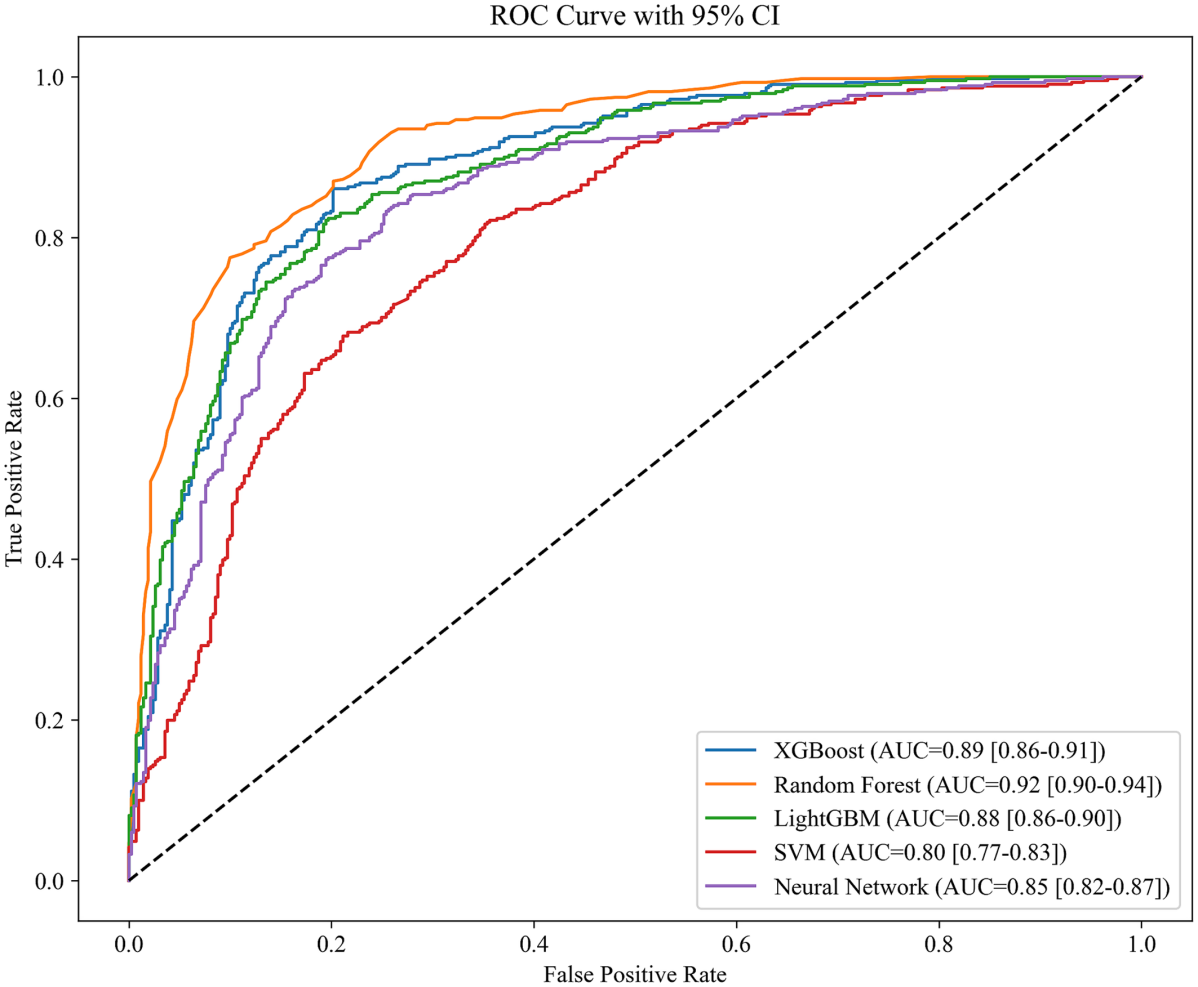
AUC results of 5 machine learning modeling algorithms.

**Figure 3:**
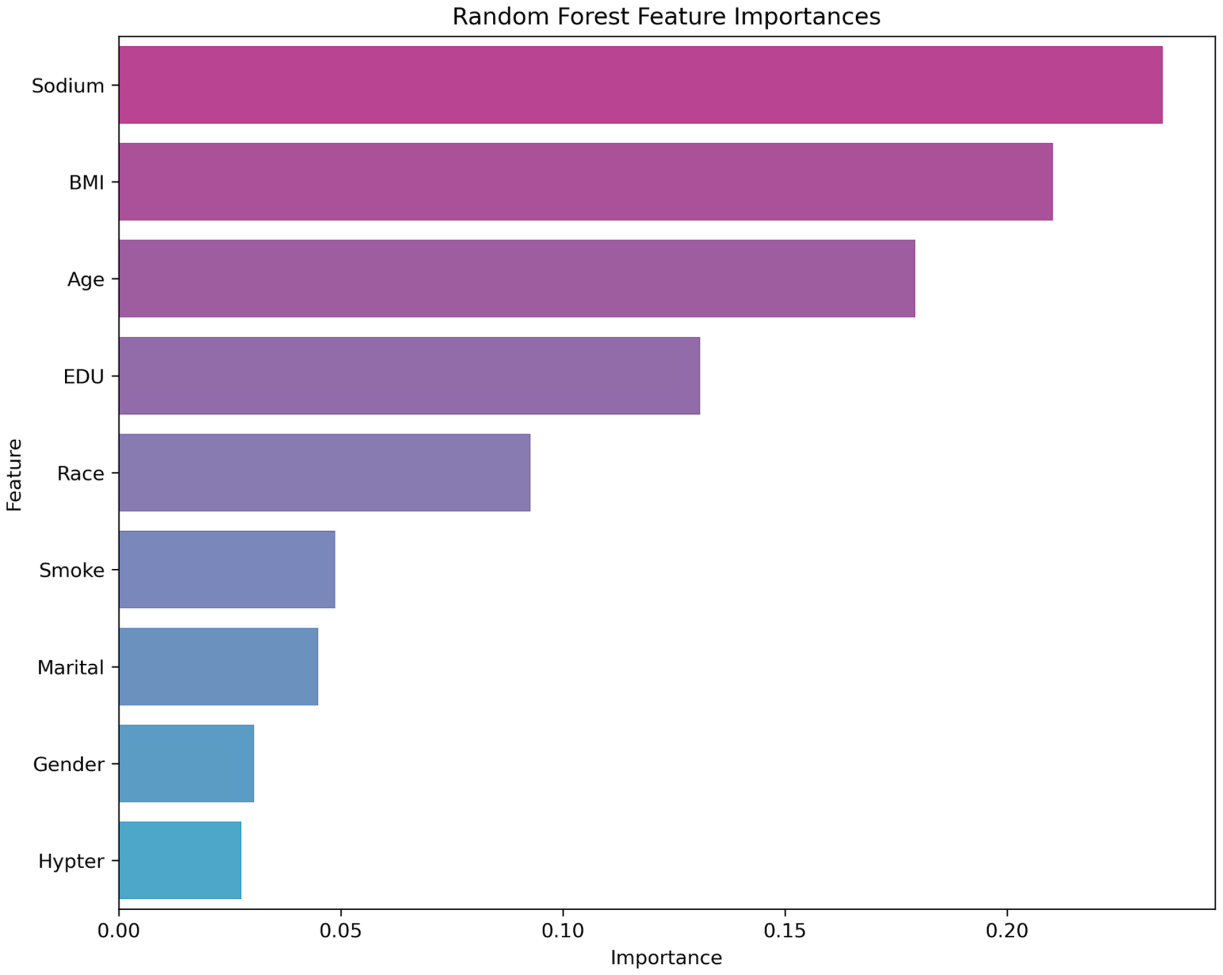
Random forest algorithm to rank all covariates.

Figure 2 shows that sodium, BMI, and age are the key variables affecting the model predictions in the random forest analysis, and are significantly more important than other features like sex, marital status, or hypertension. These results suggest that health-related factors in the prediction task, such as sodium level, BMI, and age, may have greater explanatory power for cognitive dysfunction. From the perspective of model selection, the excellent performance of the random forest model highlights its robustness in dealing with complex feature relationships and nonlinear data, particularly with an AUC value of more than 0.9, which suggests that it can differentiate between positive and negative class samples effectively. In the future, we can further analyze the contribution of its key features (see Figure 2) to the prediction or attempt model fusion to improve the performance. For SVMs and neural networks, it is necessary to optimize hyperparameters or introduce feature engineering in combination with specific data characteristics to improve their generalizability (see Figure 3).

### 3.4 Effects of HSD exposure on spatial memory in mice

The MWM test can reflect the spatial learning memory ability of mice, and the experiment was conducted in two stages: an orientation navigation experiment and a spatial exploration experiment.

#### 3.4.1 Directional navigation

**Figure 4:**
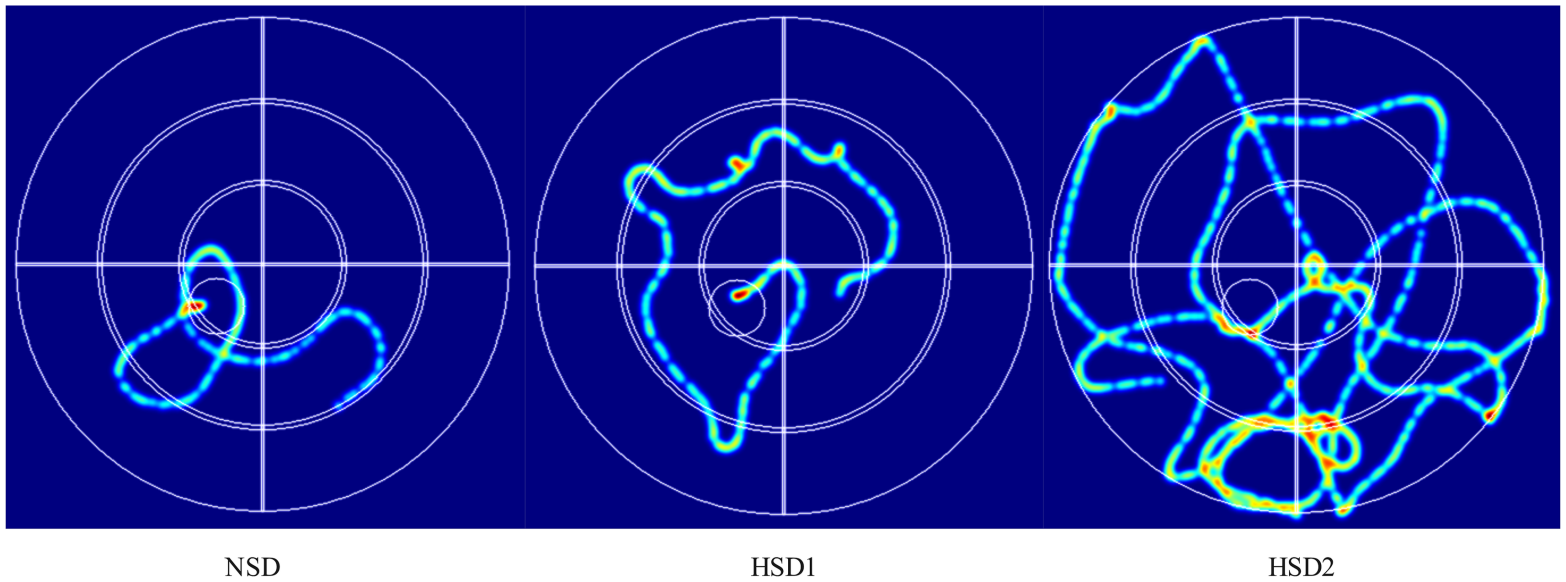
Representative inset showing trajectories in the mouse localization navigation experiment.

**Figure 5:**
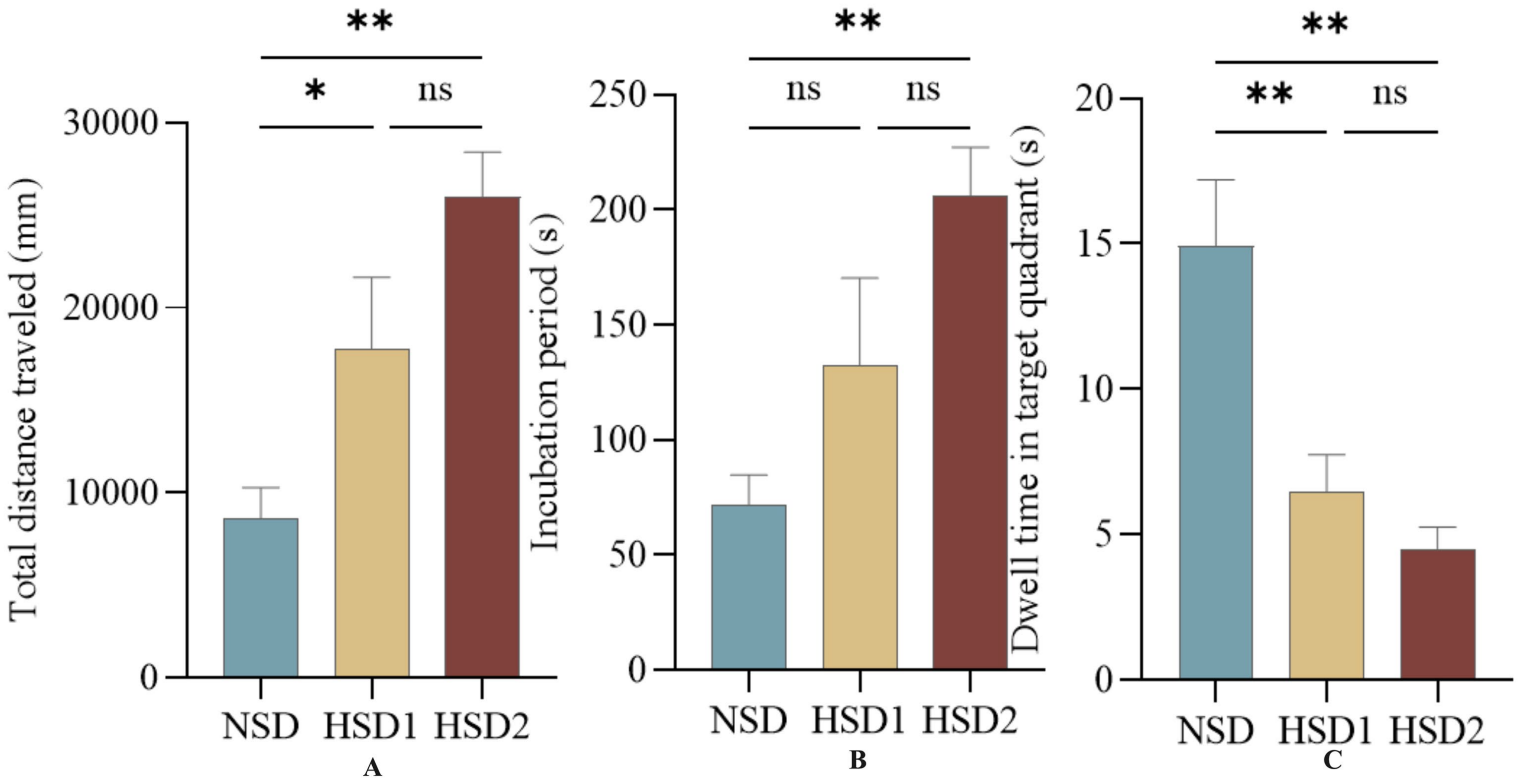
Effects of HSD exposure on spatially oriented navigation experiments in mice. **(A)** Compared with the NSD group, the HSD group was more effective in the whole trial in the total distance traveled during the test; **(B)** latency; **(C)** Dwell time in the target quadrant.

Figure 4 depicts the movement trajectories of the mice in the orientation sailing experiment during the 6-day orientation sailing experiment. The results revealed that there was a difference in the total distance traveled by the HSD 1 group compared with the NCD group (*p* < 0.05), and there was a significant difference in the HSD 2 group compared with the NCD group (*p* < 0.01) (Figure 5A). No difference (*p < 0*.01) was observed in the latency of the mice in the HSD 1 group compared with that of the mice in the NCD group. However, there was a significant difference (p < 0.01) in the latency of the HSD 2 group compared with that of the HSD group in the navigation localization experiment (Figure 5B). In addition, there was a significant difference (*p* < 0.01) in the dwell time in the target quadrant in the navigation experiment in both the HSD1/2 groups of mice and the NCD group of mice (Figure 5C).

#### 3.4.2 Space exploration experiments

**Figure 6:**
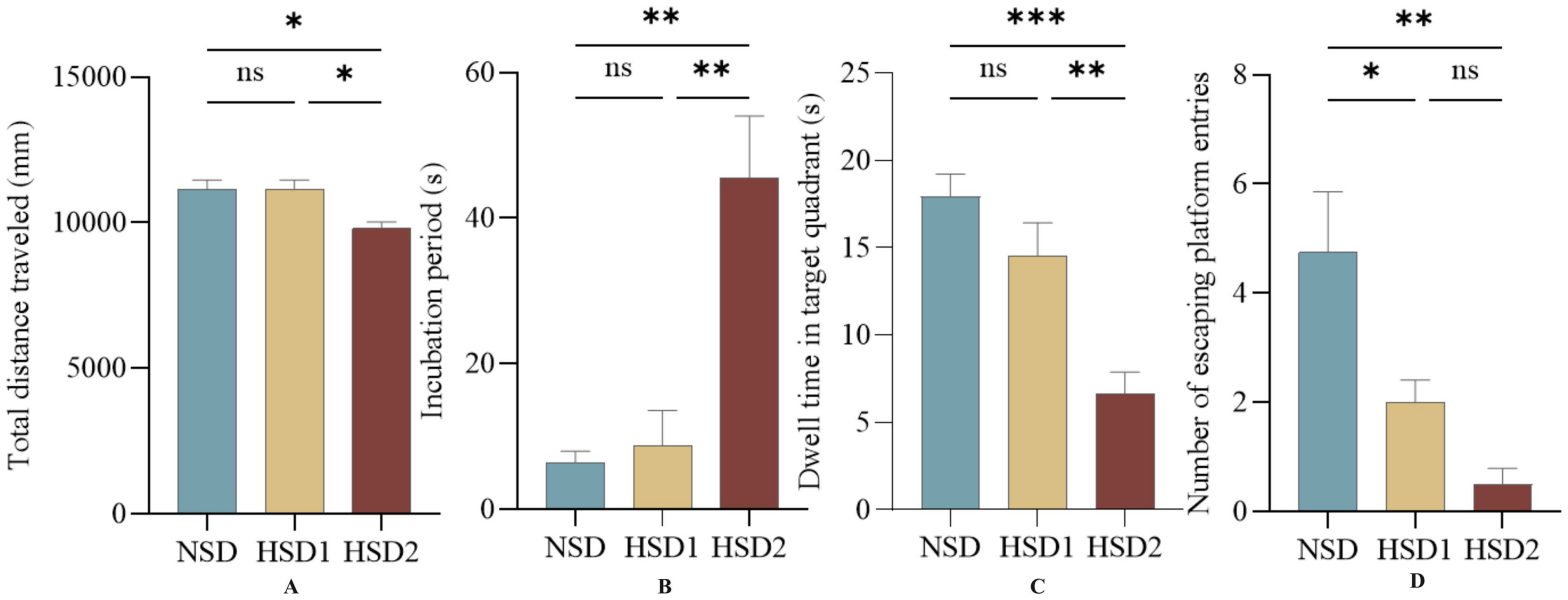
Effects of HSD exposure on spatial exploration in mice. **(A)** Total distance traveled by the HSD group throughout the trial compared with the NSD group; **(B)** latency; **(C)** dwell time in the target quadrant; **(D)** number of escape platform entries.

The results of the spatial exploration experiment revealed no difference in the total distance traveled between the HSD 1 group and the NCD group, whereas a difference was observed between the HSD 2 group and the NCD group (*p* < 0.05) (Figure 6A). There was no difference in the latency of the HSD 1 group compared with the NCD group, and there was a significant difference in the latency of the HSD 2 group (*p* < 0.01) (Figure 6B). There was no difference in the target quadrant dwell time in the space exploration experiment for the HSD 1 group compared with the NCD group, and there was a highly significant difference in the target quadrant dwell time for the HSD 2 group compared with the NCD group (*p* < 0.0001) (Figure 6C). In addition, there was a difference in the number of escape platform entries in the space exploration experiment for the HSD1 group (p < 0.05) and a significant difference in the number of escape platform entries in the space exploration experiment for the HSD2 group (*p* < 0.001) compared with the NCD group (Figure 6D).

### 3.5 Effects of HSD exposure on anxiety levels in mice

**Figure 7:**
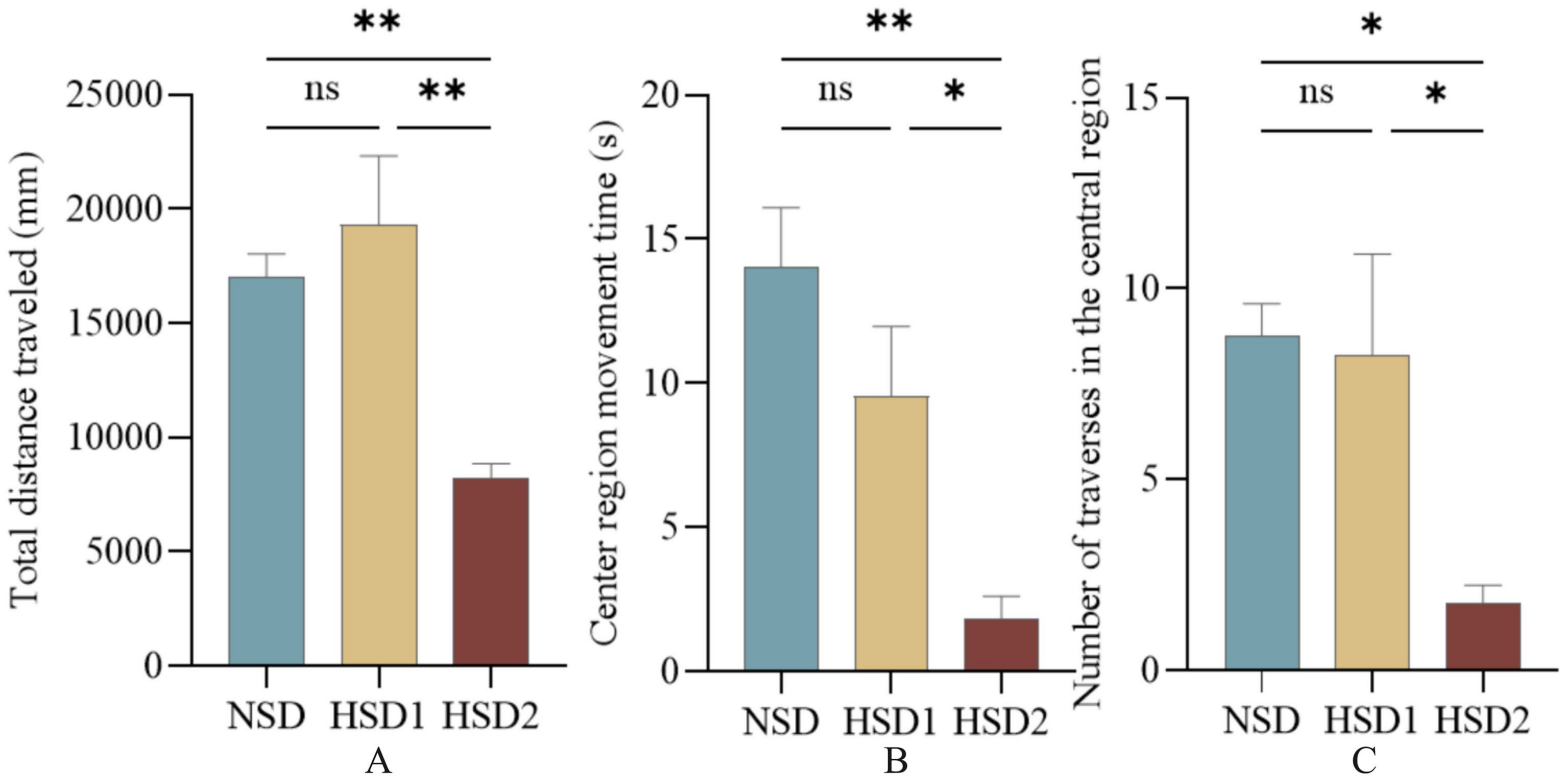
Effects of HSD exposure on mouse absenteeism. **(A)** Total distance traveled by the HSD group throughout the test period compared with the NSD group; **(B)** time of movement in the central region; **(C)** number of traversals in the central region.

In the open-field experiment, the total distance the mice traveled in the open field and the time they spent in the center reflected the ability of the mice to move independently and their anxiety level in the unfamiliar environment. The results revealed that there was no significant difference in the total distance traveled in the open field in the HSD 1 group compared with that in the NCD group, and the total distance traveled in the open field in the HSD 2 group was significantly lower (*p* < 0.01) (Figure 7A). The time spent in the center of the open field was lower (p < 0.01) in the HSD 2 group than in the NCD group (Figure 7B). In addition, the number of times that the HSD2 group crossed the center of the open field decreased (*p* < 0.05) compared with that of the NCD group (Figure 7C). This finding indicated a greater level of anxiety in the HSD group than in the NCD group.

## 4 Discussion

Cognitive dysfunction refers to a mental disorder in which an individual’s cognitive function is impaired, resulting in significant effects on daily life, social interaction, and work ability. It manifests in various forms, including memory loss, slow thinking, lack of concentration, and loss of judgment, which seriously affect the quality of life and social participation of elderly individuals. With the intensification of aging, the prevalence of mild cognitive impairment (MCI) in China is increasing annually (21). Surveys have shown that China has the world’s largest population of dementia patients (22) and that MCI can progress to different degrees in elderly individuals (23, 24). Some studies predict that the number of people with dementia in China will reach 23.3 million by 2030 and that the total cost of dementia is expected to reach US$114.2 billion by 2030 (25), placing a heavy economic and social burden on the public health system.

A high-salt diet (HSD) refers to an eating pattern characterized by sodium intake that exceeds physiological needs. According to the World Health Organization, the daily intake of sodium chloride per person should not exceed 5 g, and a value exceeding this value is defined as a high-salt diet. High-salt diets are among the most significant contributors to chronic noncommunicable diseases, accounting for more than half of diet-related deaths (26). Owing to the popularity of processed foods and fast food, the dietary intake of salt in developed and developing countries is generally high, with an average daily intake of 10–15 gNaC1 (27), which far exceeds the WHO recommended intake. Excessive salt intake can interfere with the tricarboxylic acid cycle (28), lead to insulin resistance (29), induce oxidative stress (30), etc., so high-salt diets have also been confirmed to be associated with diseases such as hypertension, cardiovascular disease, metabolic syndrome, kidney stones, osteoporosis, and cancer (13). The traditional view is that the harmful effects of high-salt diets on the brain are attributed mainly to high blood pressure, but a series of recent studies have clearly pointed out that high-salt diets are independent risk factors for cognitive impairment. A systematic analysis of 15 items related to high-salt diets and cognitive impairment revealed that high-salt diets were associated with impaired cognitive function and not with hypertension; a low-sodium diet was associated with improved cognitive performance at 6 months (31). Another epidemiological study revealed that a high-salt diet was associated with stroke, dementia, and white matter damage and was not associated with hypertension (32). Although there has been some concern about the health effects of high-salt diets, the specific effects and mechanisms of high-salt diets on brain cognition have rarely been explored, and the number of relevant studies is scarce and insufficient. With the increasing aging of the global population, the incidence of cognitive decline and related diseases, such as neurodegenerative diseases, such as Alzheimer’s disease, is increasing. In the dual context of China’s high-salt diet and the intensification of population aging, exploring the impact of a high-salt diet on brain cognitive function is highly important for the prevention and management of cognitive decline in elderly individuals. Therefore, this study uses the NHANES database to investigate the effect of salt intake on cognitive dysfunction and intends to confirm that a high-salt diet affects cognitive dysfunction.

This study included 2,367 middle-aged and older adults aged 60–80 years, and 91% of the participants had a daily sodium intake significantly higher than the recommended value of 2,300 mg/day. The distribution of sodium intake varied significantly across sociodemographic characteristics such as sex, race, education, and marital status, but BMI did not significantly differ. This suggests that the problem of high sodium intake is prevalent and closely related to the socioeconomic and cultural background of the population. However, body weight status may not be the primary driver of differences in sodium intake. Dietary sodium intake was found to be significantly and positively correlated with DSST scores and animal performance on the fluency test by weighted linear regression analysis, suggesting that high-sodium diets may specifically impair information processing speed and executive function through mechanisms such as affecting the efficiency of nerve conduction or inducing neuroinflammation. Notably, the correlation of the CERAD test was attenuated after fully adjusting the model, suggesting that it may be indirectly mediated through vascular factors. Machine learning analyses further validated these findings, with a random forest model identifying sodium intake as one of the three key predictors of cognitive impairment after age and BMI.

In the binary classification task of predicting cognitive dysfunction, this study compares the performance of five machine learning models, among which the random forest model (AUC = 0.92, accuracy = 0.83, F1 score = 0.83) performs the best, and its high robustness is attributed to the automatic capture of high-dimensional data feature interactions and the ability to resist overfitting. XGBoost and LightGBM are the second best, which reflects that the gradient XGBoost and LightGBM are the following most robust algorithms, reflecting the potential of gradient boosting algorithms in nonlinear relationship modeling, whereas the SVM and fully connected neural networks are relatively weak, which may be limited by high-dimensional data adaptability or an unoptimized network architecture. The feature importance analysis of random forests further revealed that sodium intake, BMI, and age were the core variables predicting cognitive impairment and were significantly more important than other features, such as sex and marital status. The results suggest that the random forest model can be used as an efficient clinical screening tool for early risk stratification in combination with easily accessible sodium intake and metabolism metrics. Moreover, the prominent role of sodium intake suggests its importance as an independent risk factor, which provides data support for subsequent mechanistic studies.

Animal behavioral experiments revealed that high-salt dietary exposure significantly impaired neurobehavioral function in mice in a dose-dependent manner. In the orientation navigation experiment, the total distance traveled by the HSD2 group significantly increased, the latency period increased, and the residence time in the target quadrant decreased; however, only the total distance traveled differed in the HSD1 group, suggesting that the spatial learning efficiency was weakened by high sodium intake. The spatial exploration experiment further revealed that the number of escape platform entries and the target quadrant residence time in the HSD2 group were extremely significantly lower than those in the NSD group, whereas only the number of escape distances was reduced in the HSD1 group, suggesting that the impairment of memory retention by high sodium intake was aggravated with increasing doses. In the open field experiment, the total distance moved, center dwell time, and number of traversals were significantly decreased in the HSD2 group, suggesting increased anxiety behavior and limited autonomy. The number of escapes was also decreased in the HSD1 group. The spatial memory deficits and anxiety behaviors together pointed to impaired hippocampal and prefrontal functions, suggesting that high salt may trigger multidimensional neurological dysfunction through oxidative stress, neuroinflammation, or blood–brain barrier disruption. Thus, animal experiments confirmed that a high-sodium diet directly impaired spatial memory and induced anxiety in a dose-dependent manner, providing key experimental evidence for sodium-cognition causality in population studies.

Based on the association between a high-salt diet and cognitive dysfunction and dose-dependent impairment revealed in this study, future studies should focus on deeply exploring the molecular mechanism of direct nerve damage caused by high salt intake and establish a cross-species dose–effect model to determine the key intervention window. Moreover, we will develop an accurate prediction tool that integrates sodium intake data and validates the long-term benefits of salt reduction strategies in delaying cognitive decline in a large population, ultimately driving the clinical translation of targeted interventions and public health policies. Finally, understanding how to effectively prevent and control cognitive dysfunction caused by high salt intake through drug intervention is an important area of research (33–35).

## 5 Conclusion

This study suggested that a high-salt diet is an important indicator of induced cognitive dysfunction in older adults. NHANES-based analyses highlighted a significant association between dietary sodium intake and cognitive function, emphasizing its potential role in identifying high-risk individuals. In addition, machine learning models demonstrated strong predictive performance, suggesting potential clinical applications for assessing depression risk. Experimental animal behavioral studies have also validated the causal mechanisms of these associations. Thus, the present study supports, through multidimensional evidence, that high sodium intake directly impairs cognitive function and emphasizes its importance as a target for public health intervention.

## Data Availability

The original contributions presented in the study are included in the article/Supplementary material, further inquiries can be directed to the corresponding authors.
