# Supplementary material for: Analysis of the association between dietary sodium intake and cognitive function: a NHANES-based machine learning study and animal experimental validation
Source: Front Nutr. 2025 Sep 8;12:1626651. doi: 10.3389/fnut.2025.1626651 (PMC12452095; doi:10.3389/fnut.2025.1626651)
Supplement: Supplementary file 1 [file Data_Sheet_1.docx]

**Supplementary Table 1** Sensitivity analysis

|  |  | **2.5%CI** | **97.5%CI** | **OR** |
| --- | --- | --- | --- | --- |
| Model 1 |  | 1.99 | 6.17 | 3.51 |
|  | Sodium | 1.00 | 1.00 | 1.00 |
| Model 2 |  | 140.20 | 35730.65 | 2238.16 |
|  | Age65~80 | 0.87 | 0.93 | 0.90 |
|  | Female | 1.22 | 2.62 | 1.79 |
|  | Other Hispanics | 0.58 | 1.73 | 1.00 |
|  | Non-Hispanic whites | 1.55 | 4.68 | 2.69 |
|  | Non-Hispanic blacks | 0.95 | 3.28 | 1.77 |
|  | Other races | 1.13 | 3.69 | 1.04 |
|  | married | 0.48 | 1.48 | 0.85 |
|  | Widowed/divorced | 0.34 | 1.17 | 0.63 |
|  | Sodium | 1.00 | 1.00 | 1.00 |
| Model 3 |  | 44.74 | 15310.30 | 827.67 |
|  | Age65~80 | 0.88 | 0.94 | 0.91 |
|  | Female | 1.29 | 2.97 | 0.96 |
|  | Other Hispanics | 0.54 | 1.59 | 0.92 |
|  | Non-Hispanic whites | 0.81 | 3.46 | 1.68 |
|  | Non-Hispanic blacks | 0.65 | 2.69 | 1.32 |
|  | Other races | 0.69 | 2.69 | 1.36 |
|  | married | 0.52 | 1.69 | 0.94 |
|  | Widowed/divorced | 0.40 | 1.41 | 0.75 |
|  | Congrats (on passing an exam) | 0.77 | 2.77 | 1.46 |
|  | High school and above | 1.57 | 2.77 | 3.21 |
|  | overweight (baggage, freight) | 0.64 | 1.82 | 1.08 |
|  | obese | 1.08 | 2.04 | 1.49 |
|  | Used to smoke | 0.54 | 2.26 | 1.11 |
|  | nonsmoking | 0.38 | 1.31 | 0.71 |
| Model 3 | Non-hypertensive | 0.53 | 1.20 | 0.79 |
|  | Sodium | 1.00 | 1.00 | 1.00 |
